# Creativity measured by divergent thinking is associated with two axes of autistic characteristics

**DOI:** 10.3389/fpsyg.2014.00921

**Published:** 2014-08-19

**Authors:** Hikaru Takeuchi, Yasuyuki Taki, Atsushi Sekiguchi, Rui Nouchi, Yuka Kotozaki, Seishu Nakagawa, Carlos M. Miyauchi, Kunio Iizuka, Ryoichi Yokoyama, Takamitsu Shinada, Yuki Yamamoto, Sugiko Hanawa, Tsuyoshi Araki, Hiroshi Hashizume

**Affiliations:** ^1^Division of Developmental Cognitive Neuroscience, Institute of Development, Aging and Cancer, Tohoku UniversitySendai, Japan; ^2^Division of Medical Neuroimaging Analysis, Department of Community Medical Supports, Tohoku Medical Megabank Organization, Tohoku UniversitySendai, Japan; ^3^Department of Radiology and Nuclear Medicine, Institute of Development, Aging and Cancer, Tohoku UniversitySendai, Japan; ^4^Department of Functional Brain Imaging, Institute of Development, Aging and Cancer, Tohoku UniversitySendai, Japan; ^5^Human and Social Response Research Division, International Research Institute of Disaster Science, Tohoku UniversitySendai, Japan; ^6^Smart Ageing International Research Center, Institute of Development, Aging and Cancer, Tohoku UniversitySendai, Japan; ^7^Japan Society for the Promotion of ScienceTokyo, Japan

**Keywords:** creativity, divergent thinking, empathizing, systemizing, *D* score, autistic characteristics

## Abstract

Creativity generally involves the conception of original and valuable ideas, and it plays a key role in scientific achievement. Moreover, individuals with autistic spectrum conditions (ASCs) tend to achieve in scientific fields. Recently, it has been proposed that low empathizing and high systemizing characterize individuals with ASCs. Empathizing is the drive to identify the mental status of other individuals and respond to it with an appropriate emotion; systemizing is the drive to analyze a system. It has been proposed that this higher systemizing underlies the scientific achievement of individuals with ASCs, suggesting the possible positive association between creativity and systemizing. However, previous findings on the association between ASCs and creativity were conflicting. Conversely, previous studies have suggested an association between prosocial traits and creativity, indicating the possible association between empathizing and creativity. Here we investigated the association between creativity measured by divergent thinking (CDT) and empathizing, systemizing, and the discrepancy between systemizing and empathizing, which is called *D* score. CDT was measured using the S-A creativity test. The individual degree of empathizing (empathizing quotient, EQ) and that of systemizing (systemizing quotient, SQ), and *D* score was measured via a validated questionnaire (SQ and EQ questionnaires). The results showed that higher CDT was significantly and positively correlated with both the score of EQ and the score of SQ but not with *D* score. These results suggest that CDT is positively associated with one of the characteristics of ASCs (analytical aspects), while exhibiting a negative association with another (lower social aspects). Therefore, the discrepancy between systemizing and empathizing, which is strongly associated with autistic tendency, was not associated with CDT.

## INTRODUCTION

The broadly accepted standard definition of creativity is the ability to produce work that is both novel and useful within a certain social context (Stein, 1953; Runco and Jaeger, 2012). As summarized similarly in our previous study (Takeuchi et al., 2013b), creative cognition has been essential to the development of human civilization and plays a crucial role in cultural life. Divergent thinking is defined as the generation and application of several different ideas to solve a given problem (Runco, 1990). It has been proposed as a key aspect of creative cognition (Guilford, 1967), and its strong predictive validity of creative achievement has been identified through meta-analysis (Kim, 2008).

Furthermore, the association between autistic spectrum conditions (ASCs) and scientific achievement is well known (Baron-Cohen, 2003). Recently, it has been proposed that low empathizing and high systemizing characterize ASCs. Empathizing is defined as the drive to identify the mental states of others to predict their behavior and respond with an appropriate emotion (Baron-Cohen et al., 2005). Systemizing is defined as the drive to analyze a system in terms of the rules that govern it to predict its behavior (Baron-Cohen et al., 2005). Moreover, it has been proposed that this higher systemizing underlies the scientific achievement of individuals with ASCs (Baron-Cohen, 2003). However, the results of reports on whether subjects with ASCs show higher or lower creativity measured by divergent thinking (CDT) and other types of creativity are conflicting (Rawlings and Locarnini, 2008; Claridge and McDonald, 2009; Liu et al., 2011). In addition, it is unknown whether CDT is associated with the two axes of ASCs, empathizing and systemizing, and with the discrepancy between systemizing and empathizing (called *D* score). Related to this point, CDT has been linked to certain prosocial traits, such as extraversion and cooperativeness (Chavez-Eakle et al., 2006; Takeuchi et al., 2013b); in addition, empathizing and prosocial traits are also associated (Nettle, 2007). From the perspective of neuroscience, it has been observed that understanding others (which is a part of empathizing) and self-reflection, have overlapping neural bases in the brain’s “default mode” network and overlapping cognitive bases (Saxe et al., 2006; Dimaggio et al., 2008, 2009). On the other hand, CDT, creative cognition, and empathizing have all been associated to the neural mechanisms of the default mode network (Fink et al., 2010, 2012; Takeuchi et al., 2011a,b, 2012a, 2013c, 2014b,c), suggesting the overlap of their neural bases. Considering all of these aspects, we hypothesized that higher empathizing and higher systemizing are both positively associated with CDT.

The purpose of this study was to test the abovementioned hypothesis and to investigate the association between CDT and the traits that are related to ASCs, e.g., empathizing, systemizing, and the discrepancy between systemizing and empathizing, in normal young adults. We further tested whether these associations are affected or mediated by psychometric intelligence. As described above, creative cognition plays several crucial roles in cultural life and in the development of our civilization. Thus, the psychological characteristics of individuals with higher CDT are of public interest.

## MATERIALS AND METHODS

### SUBJECTS

Data from 895 healthy, right-handed individuals (507 men and 388 women; mean age = 20.82 ± 1.84 years) were used in this study as part of an ongoing project aimed at investigating associations among brain imaging, cognitive functions, aging, genetics, and daily habits (Takeuchi et al., 2010a,b, 2011a,b,c, 2012b, 2013a, 2014a; Taki et al., 2010, 2011). All subjects were university, college, or postgraduate students or those who had graduated from these institutions within 1 year before the experiment and had normal vision. None had a history of neurological or psychiatric illness. A history of psychiatric illnesses and/or recent drug use was assessed using our laboratory’s routine questionnaire, in which each subject answered questions relating to their current or previous experiences of any list of illnesses and listed drugs that they had recently taken. Drug screening was performed to confirm that the subjects were not taking any illegal psychostimulants or antipsychotic drugs, which was one of the exclusion criteria used during the course of the recruitment. Subjects with exclusion criteria should have been excluded before they came to the lab, but if they came for some reason, they had to go back once it turned out they had an exclusion criterion. Handedness was evaluated using the Edinburgh Handedness Inventory (Oldfield, 1971). Written informed consent was obtained from each subject in accordance with the World Medical Association (1991). This study was approved by the Ethics Committee of Tohoku University. All experiments were performed at the laboratory.

### DIVERGENT THINKING ASSESSMENT

Similar to the case in our previous studies (Takeuchi et al., 2010b,c, 2011a,b, 2012a), the S-A creativity test (Society_For_Creative_Minds, 1969) was used to assess CDT. Guilford (1967) generated the draft plan and supervised the development of the test, after which the test was standardized for Japanese speakers (Society_For_Creative_Minds, 1969).

The test is used to evaluate verbal CDT (Society_For_Creative_Minds, 1969) and involves three types of tasks. Each task is preceded by 2 min of practice involving two questions with a 5-min time limit; thus, in total, the test took 30 min. This test was administered in a group setting. The first task requires subjects to generate unique ways of using typical objects (e.g., “Other than reading, how can we use newspapers?” An example answer is “We can use them to wrap things”). The second task requires subjects to imagine desirable functions of ordinary objects (e.g., “What are the characteristics of a good TV? Write down as many characteristics as possible.” An example answer is “A TV can receive broadcasts from all over the world”). The third task requires subjects to imagine the consequences of “unimaginable things” happening (e.g., “What would happen if all the mice in the world disappeared?” An example answer is “The world would become more hygienic”). Each task requires subjects to generate as many answers as possible. The S-A creativity test provides a total score, which was used in this study, as well as scores for the following dimensions of the creative process: (a) Fluency: fluency is measured by the number of relevant responses to questions and is related to the ability to produce and consider several alternatives. Fluency scores are determined by the total number of questions answered after excluding inappropriate responses or responses that are difficult to understand. (b) Flexibility: flexibility is the ability to produce responses from a wide perspective. Flexibility scores are determined by the sum of the (total) number of category types to which the responses are assigned based on a criteria table or an almost equivalent judgment. (c) Originality: originality is the ability to produce ideas that differ from those of others. Originality scoring is based on the sum of idea categories that are weighted based on a criteria table or an almost equivalent judgment. (d) Elaboration: elaboration is the ability to produce detailed ideas (Society_For_Creative_Minds, 1969). Elaboration scores are determined by the sum of responses that are weighted based on a criteria table or an almost equivalent judgment. These four dimensions correspond to the same concepts as those of the Torrance tests of creative thinking (TTCT; Torrance, 1966).

The total score is the sum of the originality score and that of elaboration in the version of the S-A creativity test (Society_For_Creative_Minds, 1969) used here. This is because the Fluency and Flexibility scores are highly correlated with those of Elaboration (Society_For_Creative_Minds, 1969). Scoring of the tests was performed by the Tokyo Shinri Corporation. However, for reference in this study, we also calculated the total score by adding the *z* scores of fluency, flexibility, elaboration, and originality.

The primary analysis was limited to the total score and did not include the score for each dimension because this score was highly correlated with that of the total as well as with each other (all correlations between the scores of any two dimensions had simple correlation coefficients >0.56). This is consistent with another group of rather similar DT tests (Heausler and Thompson, 1988), TTCT (Torrance, 1966). Heausler and Thompson (1988) concluded that the correlations among the subscales in TTCT were so high that each subscale could not meaningfully provide different information. Treffinger (1985) warned that independent interpretations of TTCT subscores should be avoided. Consistent with this notion, a previous study (Chávez-Eakle et al., 2007) that investigated the association between regional cerebral flow (rCBF) and each dimension revealed that different dimensions were correlated with rCBF in similar regions. Thus, we believe that using only the total score serves the purpose of this study.

Please refer to the appendix of our previous study (Takeuchi et al., 2010b,c) for a sample and the manner in which the tests were scored.

S-A creativity test scores are significantly correlated with various other external measures, such as various personality factors and problem-solving abilities in daily life, suggesting its ability to predict performance in everyday situations (Shimonaka and Nakazato, 2007). Furthermore, S-A creativity test scores are significantly correlated with the frequency of visual hypnagogic experiences, which in turn are correlated with the vividness of mental imagery and neuroticism (Watanabe, 1998). Furthermore, our previous study showed that S-A creativity test scores are positively correlated with extraversion, novelty seeking, motivational state, and daily physical activity level, which are consistent with the findings provided by the other measures of CDT (Takeuchi et al., 2013b).

In short, the points in this subsection were generally as described in our previous studies, which used this measure (Takeuchi et al., 2010b,c, 2011a,b, 2012a, 2013b).

### SYSTEMIZING QUOTIENT AND EMPATHIZING QUOTIENT QUESTIONNAIRES

The Japanese version (Wakabayashi et al., 2007) of the systemizing quotient (SQ)/empathizing quotient (EQ) questionnaire (Baron-Cohen et al., 2003; Baron-Cohen and Wheelwright, 2004) was administered to the subjects as in our previous studies (Takeuchi et al., 2013c, 2014b,c). The EQ score was used as an index of empathizing and the SQ score was used as an index of systemizing. This questionnaire consists of 40 items for each quotient and 20 unscored filler items. The scales consist of self-descriptive statements that are scored on a four-point scale ranging from Strongly Disagree to Strongly Agree. Half the items are worded to produce an “Agree” response and the other half a “Disagree” response. Items are randomized to avoid a response bias. Each strong systemizing/empathizing response is awarded two points, each slight systemizing/empathizing response is awarded one point, and the remaining responses are awarded zero points (i.e., each item is scored 2, 1, 0, 0); thus, yielding a range of total scores between 0 and 80 for each quotient.

The following are examples of items included in the SQ–EQ questionnaires:

“I can tune into how someone else feels rapidly and intuitively” (EQ)

“I am good at predicting how someone will feel” (EQ)

“I am fascinated by how machines work” (SQ)

“If I were buying a stereo, I would want to know about its precise technical features” (SQ)

The psychometric properties of the questionnaire are as follows. Some studies have reported that empathizing is largely independent of systemizing, but there is a weak negative correlation between them (e.g., Wheelwright et al., 2006), whereas other studies have failed to find such negative correlations (e.g., Wakabayashi et al., 2007). Individuals with ASCs have higher SQ scores and lower EQ scores than controls (Wakabayashi et al., 2007). Males have higher SQ scores than females, while females have higher EQ scores than males (Wakabayashi et al., 2006b). In addition, students studying humanities have higher EQ scores than those studying sciences, while students studying sciences have higher SQ scores than those studying humanities (Wakabayashi et al., 2006b). Furthermore, actors have higher EQ scores (Nettle, 2006). In addition, EQ is positively correlated with the size of one’s social network (Stileman, 2007) and one’s performance on a face perception task (Penton-Voak et al., 2007). The autism spectrum quotient (AQ), which is a measure of autistic traits, is well explained by a model that includes both EQ and SQ (Wheelwright et al., 2006). These findings have demonstrated the criterion-related validity of this questionnaire. The internal consistencies of EQ and SQ, which were calculated in a previous study that includes a large sample, were 0.86 and 0.88, respectively; thus, demonstrating the reliability of this questionnaire.

Some researchers may prefer performance-based cognitive measures over questionnaires. However, as far as ASCs are concerned, the validity of this questionnaire is firmly established and this tool is widely used, whereas the performance-based tools that are used to detect ASCs do not tend to work well (Montgomery et al., 2008, 2010). This may be because subjects with ASCs can use strategies and perform at a level that is comparable with that of normal subjects (Frith, 1994).

The *D* score was calculated as previously described (Goldenfeld et al., 2005). Raw SQ and EQ scores were standardized by subtracting the population mean from the score and then dividing the result by the maximum possible score: *S* = (raw SQ score – population mean of the raw SQ score)/80 and *E* = (raw EQ score – population mean of the raw EQ score)/80. For this computation, we used estimated population means (**Table 1**) derived from a large sample (*n* = 1250) of Japanese university students in a previous study (which comprised an almost equal number of males and females; Wakabayashi et al., 2007). The discrepancy between systemizing and empathizing was then quantified as *D* = (*S* - *E*)/2. The greater the *D* score in a positive direction, the stronger one’s systemizing is relative to one’s empathizing. *D* scores close to zero represent an equal drive to systemize and empathize. The *D* score is a measure that is widely used in research by leading experts in relevant areas (Goldenfeld et al., 2005; Wakabayashi et al., 2006b, 2007; Wheelwright et al., 2006; Billington et al., 2007; Lai et al., 2012). The *D* score is better at distinguishing individuals with ASCs from controls, differentiating typical males and females (Goldenfeld et al., 2005; Wakabayashi et al., 2006b, 2007; Wheelwright et al., 2006), predicting entry into physical sciences and humanities (Wakabayashi et al., 2006b; Billington et al., 2007; Focquaert et al., 2007), and predicting programming aptitude (Wray, 2007) compared with the EQ or SQ score. However, because the *D* score has components of both *S* and *E*, examining the correlates of the *D* score alone does not reveal the whole picture. Thus, we also investigated the correlates of *E* and *S* scores. One of the problems of using the difference between two values is that when the difference is calculated, the determination of the source of the variations of the value is not possible (DeGutis et al., 2013). However, in the present study, the difference in the SDs of EQ and SQ scores was not substantial (See **Table 1**). Furthermore, *z* scores of EQ and SQ scores can be used to calculate the *D* score (Wakabayashi et al., 2007), which can control for differences in the SDs of EQ and SQ scores. However, we used the present method to calculate the *D* score partly because it is more widely used (Baron-Cohen, 2003; Baron-Cohen et al., 2005; Goldenfeld et al., 2005; Wakabayashi et al., 2006a; Auyeung et al., 2009) and partly because the distribution of the *D* score calculated using the *z* scores of EQ and SQ is very similar to that calculated using the present method and it produced similar imaging findings (Lai et al., 2012).

**Table 1:** Mean, SD, and range of psychological variables among men and women.

	Men			Women		
Measure	Mean	SD	Range	Mean	SD	Range
Age	20.90	1.94	18 to 27	20.72	1.69	18 to 27
S-A creativity test [official total score (elaboration score + originality score)]	36.26	10.67	7 to 71	38.58	9.78	7 to 68
S-A creativity test (sum of *z* scores of fluency, flexibility, elaboration and originality)	-0.29	3.75	-12.22 to 10.65	0.37	3.34	-11.02 to 10.51
Empathizing	29.46	9.58	9 to 66	34.31	9.85	12 to 63
Systemizing	27.98	8.75	6 to 56	21.49	7.41	8 to 54
*D* score	0.0577	0.0712	-0.1519 to 0.2981	-0.0132	0.0719	-0.2206 to 0.1919

Briefly, this subsection’s points were generally as described in our previous studies, using this measure (Takeuchi et al., 2013c, 2014b,c).

### ASSESSMENT OF PSYCHOMETRIC MEASURES OF GENERAL INTELLIGENCE

Raven’s Advanced Progressive Matrix is one of the purest psychometric measures of general intelligence (Raven, 1998) and is often shown to be best correlated with general intelligence. Because this is the best general intelligence measure (Raven, 1998), it was used to assess intelligence. This test was used in our study to adjust for the effect of individual psychometric measures of intelligence on the psychological variables involved in this study’s hypothesis. Raven’s Advanced Progressive Matrix (Raven, 1998) contains 36 non-verbal items requiring fluid reasoning ability. Each item consists of a 3 × 3 matrix with a missing piece to be completed by selecting the best of eight alternatives. The score of this test (number of correct answers in 30 min) was used as an index of individual psychometric measure of intelligence. It was administered to determine if adjusting the effects of general intelligence alters the association between empathizing, systemizing, *D* score, and CDT.

### STATISTICAL ANALYSES

The relationships among psychological variables were investigated using multiple regression analyses and the PASW statistical software (version 18 for Windows; SPSS Inc., Chicago, IL, USA).

We investigated the associations between CDT (S-A creativity test scores) and the other psychological variables described above after correcting for the effects of age and sex. Each multiple regression analysis investigated the associations between the S-A creativity test score and one of the following: EQ score, SQ score, and *D* score after correcting for the effects of age and sex (meaning that each analysis included three covariates). Therefore, we performed three multiple regression analyses. In addition, we investigated these associations after correcting for the effects of age, sex, and the Raven’s Advanced Progressive Matrix score (meaning that each analysis included four covariates).

In all analyses, results with a threshold of *P* < 0.05, which were corrected for false discovery rate (FDR) using the two-stage sharpened method (Benjamini et al., 2006), were considered statistically significant. The correction for multiple comparisons using this method was applied to the results of the abovementioned three multiple regression analyses. FDR is the error rate in the set of comparisons that are called significant, i.e., the proportion of comparisons that are wrongly called significant. In other words, among the multiple tested results, 5% of the results that are determined to be significant using this method are not truly significant. In FDR testing, if there is truly no signal anywhere in the tested results, an FDR-controlling method has the same control as a family wise error correction. FDR-based methods are more powerful and sensitive compared with the other approaches available for multiple statistical testing (see Benjamini and Hochberg, 1995 for a full discussion; Genovese et al., 2002). We also conducted the entire analyses again, using the sum of the *z* scores for the four dimensions (fluency, flexibility, originality, and elaboration) of the S-A creativity test, instead of the official total score of the S-A creativity test (sum of the scores for elaboration and originality).

## RESULTS

### BASIC DATA

**Table 1** shows the average ± standard deviation (SD) values for age and scores for each psychological variable. EQ and SQ scores significantly negatively correlated (simple regression analysis; *P* = 0.011, *t* = 2.550, *r* = 0.085).

### ASSOCIATIONS BETWEEN PERFORMANCE ON THE DIVERGENT THINKING TEST AND PSYCHOLOGICAL VARIABLES

We investigated the association between performance of DT (S-A creativity test score) and empathizing, systemizing, and the discrepancy between these two parameters through multiple regression analyses.

A higher S-A creativity test score was significantly and positively correlated with EQ and SQ scores but not with the *D* score. The significance and insignificance of the results were unaltered by using the sum of the *z* scores for the four dimensions (fluency, flexibility, originality, and elaboration) of the S-A creativity test, instead of the official total score of the S-A creativity test (sum of the scores of elaboration and originality). The significance and insignificance of the results were not altered by the inclusion of the score on Raven’s Advanced Progressive Matrix as a covariate. For the results of all statistical analyses, please refer to **Table 2**.

**Table 2:** Statistical values from the multiple regression analyses of associations between S-A creativity test scores and cognitive variables with the covariates of age and sex.

Variables	*N*	uncorrected*P* values	FDR-adjusted*P* values	*t* value	Standardized partial regression coefficient (β)	Simple correlation coefficients (Pearson’s *r*)*
Empathizing	895	4.51*10^-5^, 3.85*10^-4^	4.73*10^-5^, 4.00*10^-4^	4.100, 3.564	0.142, 0.124	0.168, 0.147
Systemizing	895	1.54*10^-4^, 2.19*10^-3^	8.09*10^-5^,1.15*10^-3^	3.800, 3.073	0.135, 0.110	0.081, 0.065
*D* score	895	0.492, 0.446	0.1722, 0.1561	-0.687, -0.763	-0.026, -0.028	-0.076, -0.069

The left values are statistical results of analyses using the official total score of S-A creativity test (elaboration score + originality score). The right values are statistical results of analyses using the sum of *z* scores of fluency, flexibility, elaboration and originality.

*Note other statistical values are those of multiple regression analyses but simple correlation coefficients are those from simple regression analyses.

## DISCUSSION

This study investigated the associations between CDT and empathizing, systemizing, and the discrepancy between systemizing and empathizing using a large sample of normal young adults and validated psychological measures. Consistent with our hypothesis, we demonstrated that higher CDT was associated with higher systemizing, which is one of the psychological characteristics of ASCs. However, another major psychological characteristic of ASCs (lower empathizing) was associated with lower CDT. As a result, the discrepancy between systemizing and empathizing was not associated with CDT, despite the large size of our sample. These associations were unaffected by inclusion of the psychometric measures of intelligence as a covariate in the analysis model.

The present result of an association between higher empathizing and higher CDT is congruent with previous studies that reported an association between prosocial traits and higher CDT. To the best of our knowledge, this is the first time that a higher CDT score was associated with higher empathizing (drive to identify the mental states of others in order to predict their behavior and respond with an appropriate emotion, Baron-Cohen et al., 2005). The association was congruent with our previous reports on associations between other higher prosocial traits, such as higher extraversion as well as higher cooperativeness and higher CDT (Chavez-Eakle et al., 2006; Takeuchi et al., 2013b). Furthermore, a higher CDT score was previously associated with higher social skills (interpersonal emotional intelligence), which is also congruent with the present finding. Thus, the present finding, together with previous studies, supports the idea that individuals with higher CDT exhibit prosocial traits. The reasons for the association between CDT and these traits are unclear; it may be partly ascribed to the fact that both depend on widespread brain connectivity or functional properties of the network involved in social cognition (default mode network; Takeuchi et al., 2010c, 2012a, 2013c, 2014b). However, these are speculations and future studies may need to investigate the details of these associations.

This study’s results might provide insight into problem solving in the everyday lives of individuals with ASCs. In this study, CDT was positively associated with empathizing. Furthermore, CDT is associated with the ability to solve problems in everyday life (Shimonaka and Nakazato, 2007), and individuals with ASCs have deficits solving these problems despite higher systemizing, which is apparently likely to contribute to problem solving (Baron-Cohen, 2003). There could be numerous sources for this deficit, including empathizing and dysexecutive problems in individuals with ASCs. But perhaps, given the present results, lowered CDT due to lower empathizing might be one source of such deficits and indirectly hinder the adaptivity of individuals with ASCs.

The present results suggest that autistic tendency is not associated with CDT, because empathizing and systemizing positively contributed to CDT. To the best of our knowledge, this is the first time that a higher CDT score was associated not only with higher empathizing but also with higher systemizing. By analyzing the system, subjects with higher systemizing may be able to generate effective ideas better. The effect sizes appeared to be equal. Low empathizing and high systemizing are two essential components that are considered to characterize ASCs (Baron-Cohen, 2004). The higher systemizing observed in individuals with ASCs has been suggested to underlie the scientific achievement of these individuals (Baron-Cohen, 2004). However, findings of an association between ASCs and CDT are not consistent and remain elusive, as described in the Section “Introduction.” The present results suggest that at least one of the reasons for the lack of a robust association between ASCs and CDT is the fact that while higher systemizing contributes to higher CDT, lower empathizing works in the opposite manner, and there are no effects of the association between ASCs (which is very highly predicted by the *D* score, Wheelwright et al., 2006) and CDT.

In this research, we measured empathizing and systemizing through questionnaires, believing them to be the method developed by leading experts on autism (Baron-Cohen, 2003). As for ASCs, perforamnce-based measures are known for their pitfalls because subjects with apparent dysfunctions do not manifest poorer performance than others (Montgomery et al., 2008, 2010), possibly due to the subjects developing alternative strategies (Frith, 1994). Nonetheless, questionnaire-based measures of cognition are also historically known for their pitfalls. For example, the correlation between the performance-based measures for the emotion-related competence and the questionnaire-based measures for emotion-related competence are moderate (for example, in the case of ability emotion intelligence and trait emotion intelligence *r* = 0.46; Bar-On, 2000). Moreover, while performance-based measures tend to correlate with general intelligence (Lam and Kirby, 2002), questionnaire-based measures tend to correlate with other questionnaire-based measures as well (Gardner and Qualter, 2009). In other words, performance-based measures and questionnaire-based measures reveal different psychological characteristics. Particularly, in severe brain injuries or neuronal degenerations, subjects lack insight into their symptoms or show deficits in metacognition (Prigatano, 1991), and this deficit is associated with other clinical problems (Lysaker et al., 2008, 2010, 2011). Although, in non-clinical subjects, this situation may be less of an issue. For example, in the evaluation of dysexecutive symptoms through questionnaires, in clinical samples, others estimate dysexecutive symptoms more severely than the subjects themselves; conversely, in non-clinical samples, subjects estimate their problems more severely (Burgess et al., 1998). This suggests that only among clinical samples do subjects underestimate the severity of their dysexecutive problems. In particular, patients with schizophrenia are known for their deficits in the core components of empathy (Derntl et al., 2009). On the other hand, patients with schizophrenia are also known for their deficits in metacognition and self-reflection (Dimaggio et al., 2009). In fact, self-reflection and understanding others’ minds are partly overlapping (as well as distinct) cognitive and neural bases (Saxe et al., 2006; Dimaggio et al., 2008). Possibly then, some normal adults with severe deficits in insight into themselves may have simultaneous deficits in empathetic competence, but do not notice. Thus, in the future, it would be perfect if we could evaluate empathetic competence (or systemizing, too) through semistructured interviews (Lysaker et al., 2002), performance-based measures using audiovisual stimuli, and online social interactions in order to obtain higher ecological validity in real life (Dziobek, 2012), too. Another possibility is by performing evaluations from close acquaintances, along with self-reported questionnaires. Particularly, in related investigations among subjects with severe neuronal injuries or neuronal degenerations, who are likely to have substantial deficits in metacognition and insight into their conditions, the nature of the evaluations used should be carefully considered.

There was at least another limitation to this study, which was common to our previous studies and other studies that use college cohorts (Song et al., 2008; Jung et al., 2010; Takeuchi et al., 2010b,c, 2011b; Wei et al., 2013). As previously described (Takeuchi et al., 2012a), we used young healthy subjects with high educational backgrounds. Limited sampling of the full range of intellectual abilities is a common hazard when sampling from college cohorts (Jung et al., 2010). Whether our findings would also hold across the full range of population samples and a normal distribution must be determined using larger and more representative samples. Focusing on highly intellectual subjects was certainly warranted for the purpose of this study, given the association between higher intelligence and higher CDT among subjects with normal and inferior intelligence (Sternberg, 2005). In addition, similar to our previous studies of CDT, we only used the verbal DT test as a measure of DT. Thus, our interpretation may have certain limitations in terms of generalization regarding this aspect as well, although a previous study that used both verbal and figural DT tests showed that the psychological characteristics of both tasks in terms of the association with personality traits are quite similar (Chavez-Eakle et al., 2006).

Creative cognition is important in our cultural and everyday life. Our findings showed that higher systemizing, which is one of the major characteristics of ASCs, was associated with higher CDT, whereas lower empathizing, which is another characteristic of ASCs, was associated with lower CDT. Therefore, the discrepancy between systemizing and empathizing, which is strongly associated with autistic tendency, was not associated with CDT.

## Conflict of Interest Statement

The authors declare that the research was conducted in the absence of any commercial or financial relationships that could be construed as a potential conflict of interest.
